# A rare and reversible case of heart failure—Hypocalcemia due to hypoparathyroidism

**DOI:** 10.1002/ccr3.2397

**Published:** 2019-08-29

**Authors:** Josiana de Oliveira Martins Duarte, Paula Maria Lobato Pestana Pereira, Ana Sofia Gonçalves Sobral, David Campoamor Durán, Ana Isabel da Silva Fernandes, Henrique José Barrelas Rita, José António de Sousa e Costa

**Affiliations:** ^1^ Internal Medicine Service Hospital Litoral Alentejano Santiago Do Cacém Portugal; ^2^ Intensive Care Unit Hospital do Litoral Alentejano Santiago Do Cacém Portugal

**Keywords:** heart failure, hypocalcemia, hypoparathyroidism, left ventricular dysfunction

## Abstract

Physicians should look more carefully for the potential reversible causes of acute heart failure, namely hypoparathyroidism. The recovery of left ventricular function with the treatment of hypoparathyroidism underlines the importance of calcium and the reversibility of this type of cardiomyopathy.

## 1 INTRODUCTION

Although the role of calcium in excitation‐contraction and myocardial relaxation is well established,^1^, ^2^, ^3^ presentation as congestive heart failure due to hypocalcemia is rare. In this article, we report a case of a patient presenting at the emergency department with acute congestive heart failure, without a history of underlying myocardial disease. In this case, there was persistence of left ventricular dysfunction for a long period of time after normalization of serum calcium level.

## 2 CASE DESCRIPTION

We present a case of a 54‐year‐old woman with no previous history of cardiac events who presented at the emergency department of our hospital for history, with about 1 week of evolution, of dyspnea, persistent cough, and fatigue for progressively smaller efforts. Of the known personal antecedents, hypertension well controlled, hypercholesterolemia, hypothyroidism under medication and well controlled, an recent diagnosis (< 1month) of biocular cataracts.

At the initial objective examination, he breathed at 35 breaths per minute, and the heart rate was 109/min in sinus rhythm on the monitor, with a blood pressure of 136/83 mm Hg. Pulmonary auscultation showed crackling fervor in the lower 2/3 of both hemithorax. Cardiac auscultation showed hypofonese of S1 and S2. At the abdominal and neurological summary examination, no alterations of relief were detected. The electrocardiogram showed complete left bundle branch block (we do not know whether old or new), but with normal cardiac enzymes.

Due to the presence of severe respiratory distress with the use of accessory muscles, conditioning respiratory acidosis, she was intubated orotracheally. Initial head echocardiography suggested marked dilation of the left heart chambers. From the blood analytical evaluation performed, normal hemoglobin of 11.9 g/dL was found and sodium ion (140 mmol/L), potassium (3.8 mmol/L) normal magnesium (1.7 mg/dL), Phosphate(6.1 mg/dL) and hypocalcemia 0.61 mmol/L (Table 1). After this initial evaluation, she was transferred to the intensive care unit of our hospital.

**Table 1:** Plasma values of electrolytes upon admission and after the first week of treatment

Electrolyte (total plasma values)	Upon admission	After the first week of treatment	Normal values
Calcium	0.61 mmol/L	0.93 mmol/L	1.15‐1.32 mmol/L
Potassium	3.8 mmol/L	3.6 mmol/L	3.6‐5.1 mmol/L
Sodium	140 mmol/L	143 mmol/L	136‐144 mmol/L
Magnesium	1.7 mg/dL	1.8 mg/dL	1.8‐2.5 mg/dL
Phosphate	6.1 mg/dL	4.1 mg/dL	2.5‐4.6 mg/dL

**Figure 1:**
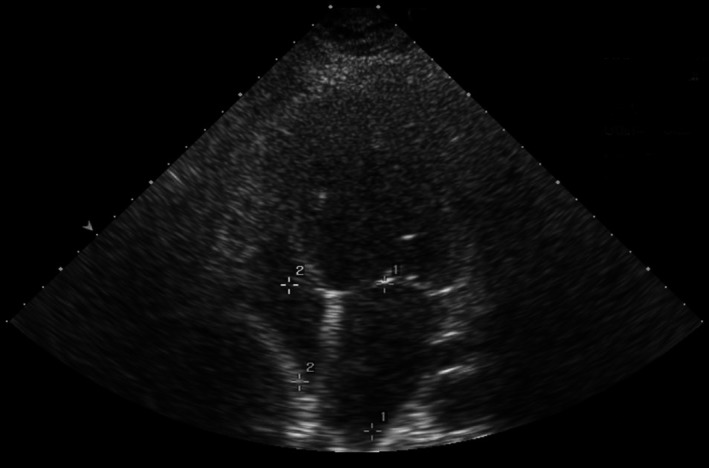
Echography at eighth day after calcium gluconate therapy, with enlargement of left heart chambers

During ICU internment, it was immediately started administration of calcium gluconate 1 g every 6 hours. Probably due to improved cardiac function under calcium therapy, anticongestive measures, and other prognostic modifying heart failure therapies, it was possible to extubate her on the sixth day of hospitalization and at eighth day, she was transferred to the intermediate care unit. The echocardiography at eighth day revealed an ejection fraction of 26% and enlargement of left heart chambers (Figure 1).

On suspicion of hyperparathyroidism due to persistent hypocalcemia and hyperphosphatemia, parathyroid hormone (PTH) titration was requested, with a result of 00.90 pg/mL (normal value 12‐88). Initially, the patient was treated with intravenous calcium supplementation and was subsequently replaced by oral calcium supplements (calcium carbonate, 1 g per day) and calcitriol (0.25 mcg/d).

From the picture of acute left heart failure, the patient was still submitted to catheterization that was normal.

Due to the young age of the patient, tests were performed to exclude the major autoimmune diseases that could mimic this type of disease, namely antinuclear antibodies, rheumatoid factor, sedimentation rate, and antiparathyroid antibodies that were all normal. Vitamin D was normal, and the viral panel was negative (hepatitis B and C and human immunodeficiency virus).

The CT scan of the neck revealed that the parathyroid glands have not been unequivocally visualized which is very probably in relation to the reduced volumetric expression of the organs. The PET scan showed examination without highlighting focal hypermetabolic lesions, namely suspicion of primary neoplastic etiology.

After 30th days of internment, she was discharged with a calcium of 1.15 mmol/L.

Based on the laboratory and echocardiographic findings, a diagnosis of heart failure due to hypocalcemia was suspected.

After 10 months of treatment with calcium supplements, the patient was reevaluated by the cardiologist who opted for the implantation of a resynchronization device as primary prevention due to the low ejection fraction. At that time, the ejection fraction was 35%.

Eleven months later, in an internal medicine reassessment study, the patient was asymptomatic without any symptom or sign of heart failure and the echocardiogram showed a significant improvement in cardiac function with ejection fraction that was of 68.6% and normal left ventricular dimensions. Thus, with the frank improvement of the ejection fraction after correction of hypocalcemia, the hypothesis of heart failure due to hypocalcemia was corroborated.

## 3 DISCUSSION

The crucial role of calcium ions in cardiac contraction and relaxation has been amply demonstrated in the literature.^4^

Hypocalcemia is a rare but treatable and reversible cause of dilated cardiomyopathy and therefore should be investigated in all unexplained cases of severe left ventricular dysfunction.^5^, ^6^ Primary hypoparathyroidism (HPTP) is characterized by an abnormally low level of secretion of parathyroid hormone (PTH), a hormone that plays a key role in calcium homeostasis.

In this case, we report that after 2 months of treatment with calcium supplements the ejection fraction remained depressed; however, what is described in the literature is that the recovery of left ventricular function can take up to 6 months in heart failure due to hypoparathyroidism.

After 1 year of treatment, our patient recovered ejection fraction (which was already normal) as well as disappearance of the left ventricular dilatation verified in the initial echocardiogram. Thus, the diagnosis of heart failure secondary to hypoparathyroidism was confirmed. The patient should maintain oral supplementation with calcium and calcitriol ad eternum.

## CONFLICT OF INTEREST

None declared.

## AUTHOR CONTRIBUTIONS

Department of Internal Medicine, Hospital of Litoral Alentejano, primary author: assumed responsibility for the publication, making sure that the data are accurate, that all deserving authors have been credited; was responsible for data collection and analysis; monitoring the progress of the disease and responsible for the literature review and final approval, submitting revisions and final version, and communicating with editors. Department of Internal Medicine, Hospital Do Litoral Alentejano Co‐authors: was coresponsible for data collection and analysis and contributed greatly to the case presentation section in the manuscript and to the discussion section of the manuscript.
